# A Rare Case of Lemierre's Syndrome Caused by Streptococcus Intermedius, Presenting as an Epidural Abscess

**DOI:** 10.7759/cureus.7787

**Published:** 2020-04-22

**Authors:** Anupama B K, Tarvinder Gilotra, Casey Tymko, Zaid Siddique, Ambika Eranki

**Affiliations:** 1 Internal Medicine, State University of New York (SUNY) Upstate Medical University, Syracuse, USA; 2 Infectious Disease, State University of New York (SUNY) Upstate Medical University, Syracuse, USA; 3 Anesthesiology, State University of New York (SUNY) Upstate Medical University, Syracuse, USA; 4 Radiology, State University of New York (SUNY) Upstate Medical University, Syracuse, USA

**Keywords:** lemierre's syndrome, streptococcus intermedius, epidural abscess, internal jugular vein thrombosis

## Abstract

Lemierre's syndrome is a rare but life-threatening condition characterized by an oropharyngeal infection typically secondary to Fusobacterium necrophorum resulting in septic thrombophlebitis of the internal jugular vein. Streptococcus intermedius is a particularly rare cause of Lemierre's syndrome with only a few cases reported in the literature. Here we describe a rare case of Lemierre's syndrome, caused by Streptococcus intermedius, likely secondary to an odontogenic infection, found to have a cervical epidural abscess with concomitant large retropharyngeal and prevertebral abscesses on presentation, in whom the clinical course was further complicated by an extensive cerebral venous sinus thrombosis. However, despite grave complications, early diagnosis and appropriate emergency management including intravenous antibiotics and surgical intervention led to a successful recovery, thus demonstrating that aggressive measures can potentially lead to a favorable outcome.

## Introduction

Lemierre's syndrome (LS), first described by French bacteriologist Andre-Alfred Lemierre, is characterized by an oropharyngeal infection resulting in septic thrombophlebitis of the internal jugular vein (IJV) followed by septic embolization [1, 2]. In 1936, Lemierre reported twenty young, healthy adult patients initially diagnosed with pharyngotonsillitis and peritonsillar abscesses who subsequently developed neck swelling and tenderness secondary to septic thrombophlebitis of the IJV with metastatic abscesses and anaerobic septicemia. In this era, the syndrome demonstrated a particularly high rate of mortality, with death occurring in eighteen of these twenty patients [3, 4]. Following the introduction of the antibiotics, LS has often been considered to be a forgotten syndrome [2, 4]. This syndrome, however, has been reported more frequently in the last twenty years, a phenomenon that has been attributed to increased awareness, increased availability of diagnostic modalities such as computed tomography (CT) and magnetic resonance imaging (MRI), and increasing antibiotic stewardship. Indeed, if fewer patients are aggressively treated for bacterial infections, then there is an increase in syndrome susceptibility [1, 5-7]. Nevertheless, LS is very rare in developed countries with an estimated incidence of one case per million per year [5, 7]. In the pre-antibiotic era, LS was associated with a case mortality rate of 32% to 90% with embolic events in 25% of patients and endocarditis in 12.5% of patients. LS continues to be a potentially life-threatening syndrome with studies in the modern era, reporting mortality rates from 0%-18% [2, 4, 5, 8].

The most common pathogen associated with LS is Fusobacterium necrophorum (F. necrophorum). Up to one-third of patients demonstrate a polymicrobial infection composed of anaerobic streptococci and other gram-negative anaerobes. Other etiological agents such as Staphylococcus, Enterococcus species, Klebsiella, and Proteus have also been isolated [4, 5]. Tonsillitis is the most common primary infection (87.1%), followed by mastoiditis (2.7%) and odontogenic infections (1.8%) [8]. Subsequent to an alteration in the pharyngeal mucosa caused by viral or bacterial pharyngitis, the pathogenic organism can penetrate the mucosal surfaces and locally invade the lateral pharyngeal space resulting in septic thrombophlebitis of the IJV. Thrombosis may then propagate from the IJV inferiorly into the subclavian vein or superiorly into the cavernous, sigmoid, or transverse sinuses. Meningitis may also complicate up to 3% of cases. Metastatic infections following IJV thrombophlebitis occurs in 63%-100% of patients. The most common sites of the metastatic infection are the lungs, followed by major joints. Metastatic infections of the liver, muscle, pericardium, brain, and skin have also been described [4]. Complications such as mediastinitis, epidural or spinal abscess, and carotid thrombosis are rare but severe [7].

Streptococcus intermedius (S. intermedius) is a gram-positive microaerophilic coccus that is a normal flora of the oral cavity, respiratory tract, and gastrointestinal tract. It is a viridans streptococcus and it, along with Streptococcus anginosus and Streptococcus constellatus, belongs to anginosus group formerly known as the Streptococcus milleri group. These three organisms are unique among viridans streptococci because they are pyogenic. S. intermedius is the most pathogenic of the three and most likely to lead to abscess formation. These abscesses can occur in the liver, brain, skin, and heart valves, even in immunocompetent patients [9]. Here we describe a rare case of LS caused by S. intermedius, likely secondary to odontongenic infection, presenting with an extensive cervical epidural abscess.

## Case presentation

A 37-year-old male with a past medical history significant for a seizure disorder and antiepileptic medication noncompliance presented to the emergency department (ED) complaining of an inability to void urine for three days. Per patient history, there was one episode of a breakthrough seizure two weeks prior to the presentation during which a fall was sustained resulting in minor facial injuries. At that time the patient presented to the ED where CT scan of the head and maxillofacial structures were unremarkable. The patient was discharged home. Following discharge, the patient experienced two weeks of worsening headaches, bilateral neck pain aggravated by a head and neck rotation, low-grade fever, fatigue, decreased appetite, difficulty swallowing, and right upper extremity weakness. He again presented to the ED after he was unable to void for three days. During this presentation, the patient denied recent dental procedures, pharyngeal infection, intravenous drug abuse, previous history of deep vein thrombosis, lower extremity weakness, or bowel dysfunction.

Initial physical exam demonstrated an oral temperature of 101 degrees Fahrenheit with otherwise normal vital signs, multiple healing scabs on the face, occipital and posterior auricular lymphadenopathy, decreased range of motion of the neck, and decreased right upper extremity strength. No edema, erythema, or palpable masses of the neck were noted. An oropharyngeal exam revealed poor dentition and gross decay of all right posterior maxillary teeth. A bladder scan revealed an abnormally elevated post-void residual volume of urine. Foley catheterization of the bladder was performed with an immediate urine output of one liter. Laboratory testing revealed a neutrophilic leukocytosis of 24,600 cells per microliter, an elevated C-reactive protein of 208.9 milligrams per liter, and an elevated erythrocyte sedimentation rate of 41 millimeters per hour. The platelet level, hemoglobin and hematocrit, comprehensive metabolic panel, and lactic acid level were all within normal limits and the serological test for HIV also returned negative. In the setting of fever, headache, right upper extremity weakness, and acute urinary retention, emergent head, and neck imaging were performed. Blood cultures were drawn and broad-spectrum intravenous empirical cefepime, vancomycin, and metronidazole were initiated.

**Figure 1:**
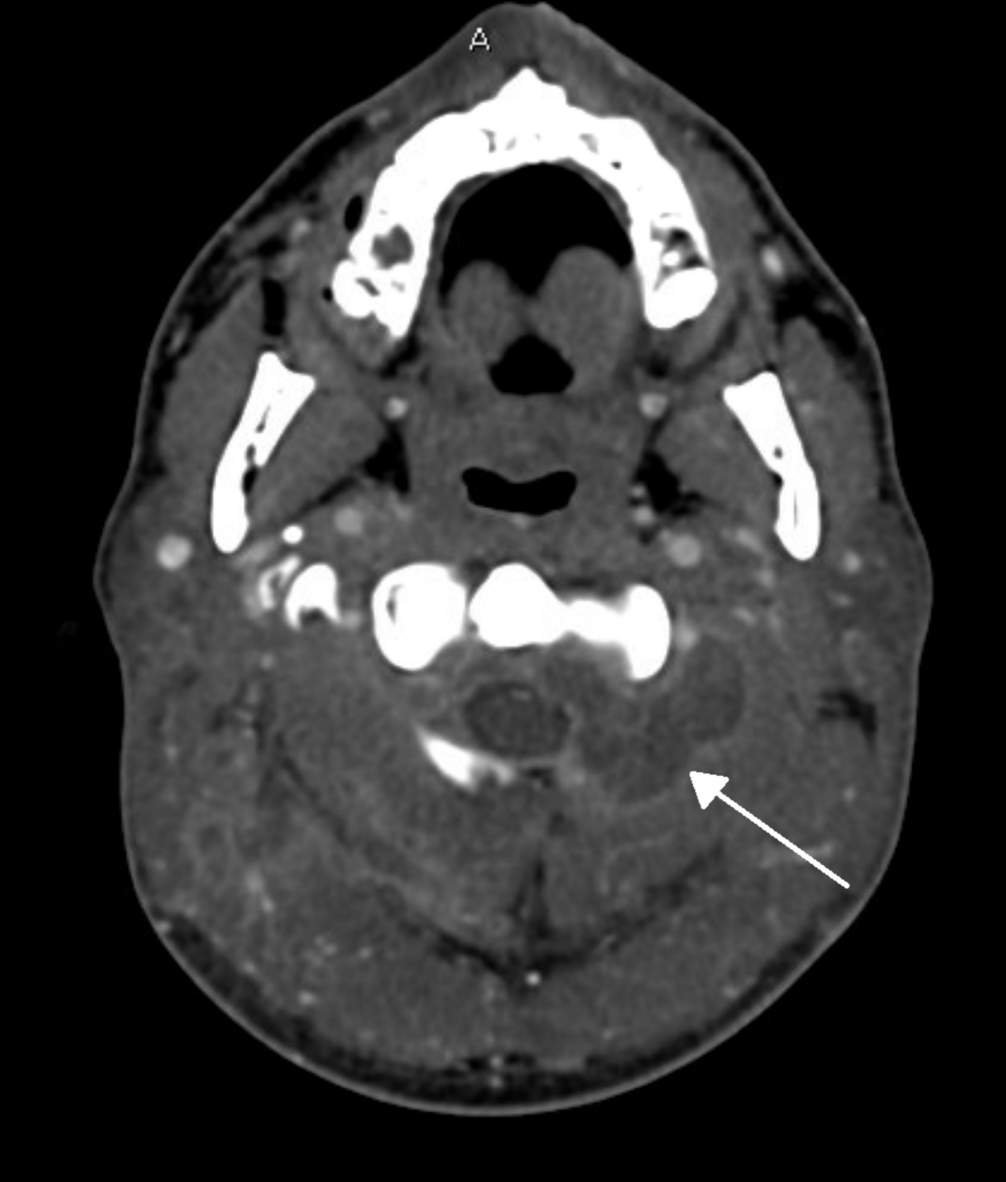
Axial CT angiography of the neck showing multiloculated abscess Image showing multiloculated abscess (arrow) extending into the left C1-C2 neuroforamina causing partial effacement of the anterolateral thecal sac.

CT angiography of the neck demonstrated peripherally enhancing fluid collections, consistent with abscesses, located within the epidural, retropharyngeal, and prevertebral spaces. Specifically, a 3.0 x 3.0 x 4.6 cm right retropharyngeal fluid collection was demonstrated causing significant mass effect with partial effacement of the hypopharynx and supraglottic airway. Additionally, a 2.7 x 3.2 x 1.5 cm collection was noted within the left paravertebral musculature with extension through the left C1-C2 neural foramina and partial effacement of the left anterolateral thecal sac, compatible with an epidural abscess (Figure 1).

**Figure 2:**
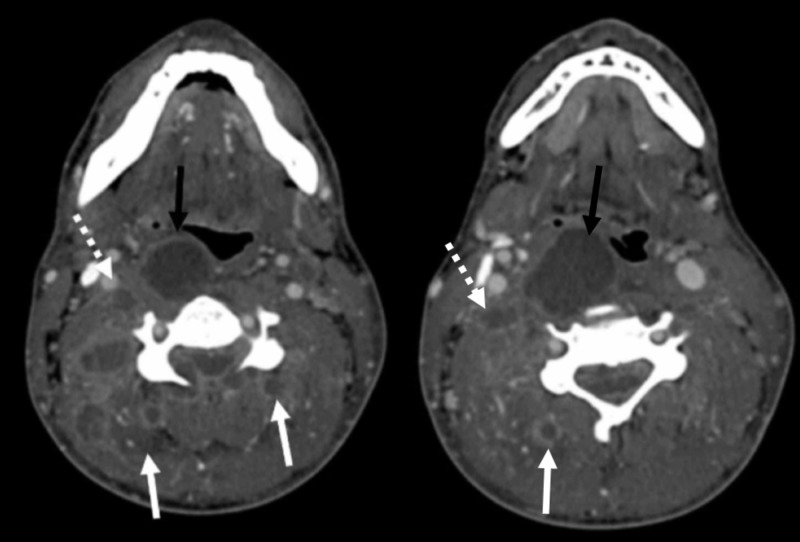
Axial CT angiography of the neck showing numerous peripherally enhancing fluid collections Image showing numerous peripherally enhancing fluid collections scattered throughout the cervical soft including the retropharyngeal space (black arrow), prevertebral (dashed arrow) and bilateral paravertebral spaces (white arrows).

Lastly, a 1.6 x 3.6 x 6.0 cm multiloculated collection was noted in the right prevertebral space with extension to the level of the thoracic inlet, without evidence of direct extension into the mediastinum (Figure 2).

**Figure 3:**
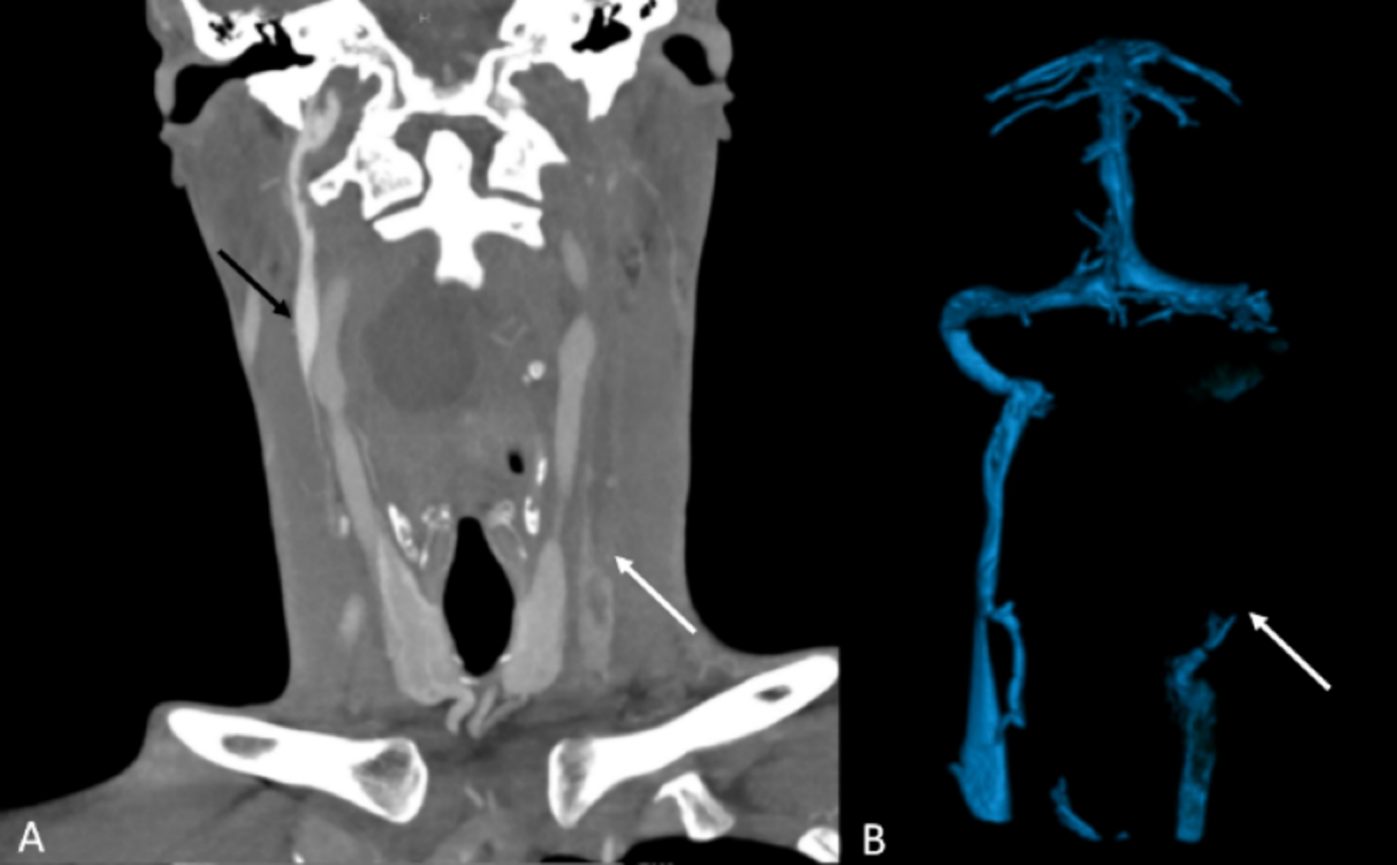
Coronal CT angiography of the neck and 3D CT reconstruction of the venous system (A) Coronal CT angiography of the neck shows complete occlusion of the left jugular vein with thrombus extending cephalad to the sigmoid sinus (white arrow) and patent right IJV (black arrow), (B) 3D CT reconstruction of the venous system demonstrates complete occlusion of the left IJV (white arrow). IJV - internal jugular vein

In addition to the noted abscesses, a large occlusive thrombus originating in the left sigmoid sinus was demonstrated with extension inferiorly throughout the proximal course of the left IJV (Figure 3).

Of note, the CT thorax showed no evidence of pulmonary abscess.

**Figure 4:**
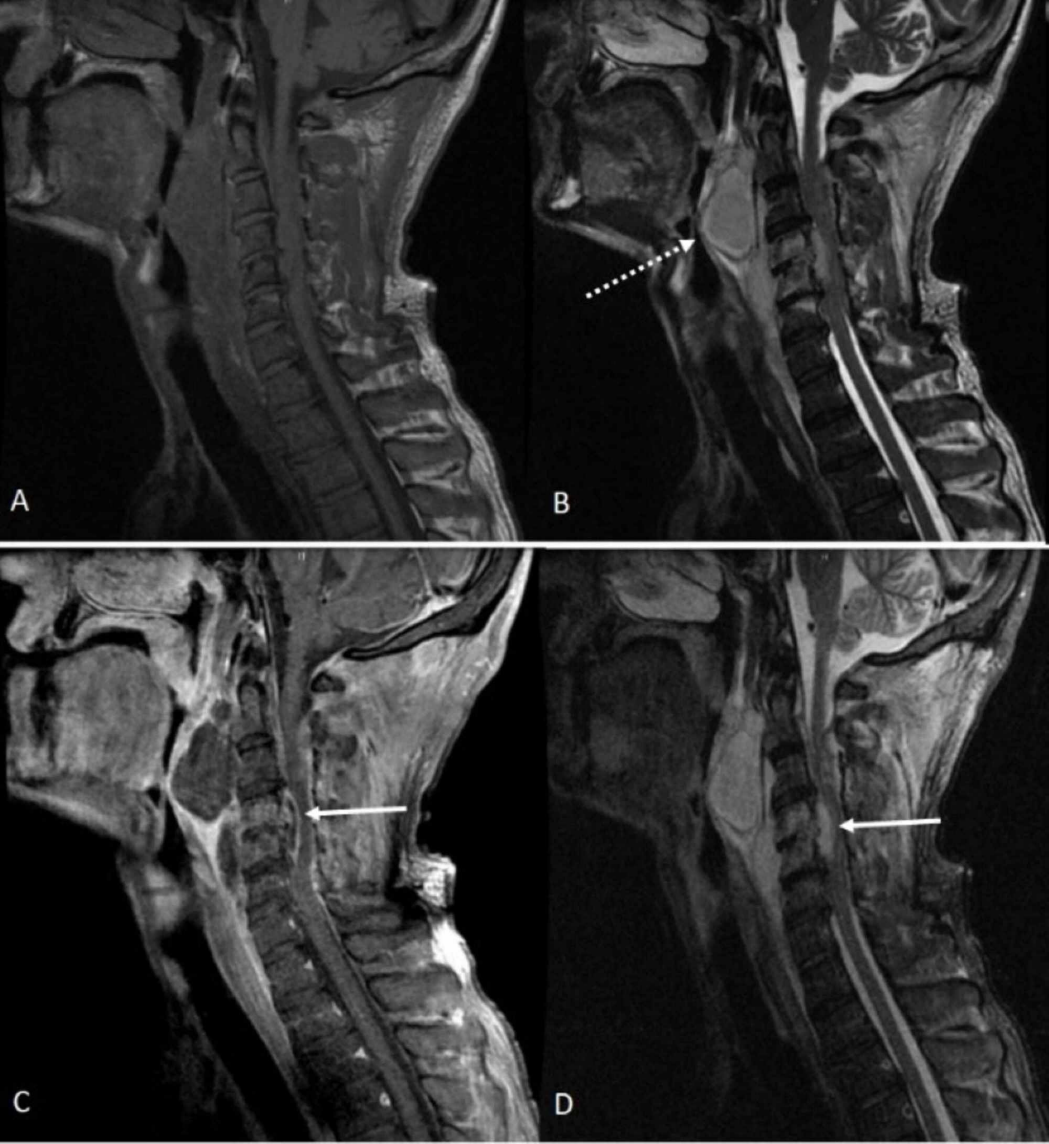
MRI of the cervical spine MRI of the cervical spine with and without contrast, sagittal sequences (A) T1, (B) T2 shows retropharyngeal abscess (dashed arrow), (C) Gadolinium-enhanced T1 shows heterogeneous enhancement in the epidural space representing an epidural abscess circumventing the spinal cord at C4-C5 (solid arrow) and hyperintensity within the C4-C5 vertebral bodies representing discitis/osteomyelitis complex, (D) short TI inversion recovery (STIR) shows epidural abscess and discitis/osteomyelitis complex at C4-C5.

The ears, nose, and throat (ENT), vascular surgery, and neurosurgery services were consulted. ENT attempted a bedside aspiration of the retropharyngeal abscess but was unsuccessful. Vascular surgery recommended treatment of the IJV thrombus with intravenous anticoagulation. Intravenous heparin was immediately initiated at this time. Neurosurgery recommended an emergent MRI. This MRI was performed and the results supplemented the findings of the previous CT scan. Specifically, a large right retropharyngeal abscess spanning from the C2 to C4 levels was demonstrated. A large left paravertebral abscess was noted with extension to the left C1 to C2 neural foramina, into the epidural fat, with partial effacement of the anterolateral thecal sac, without compression of the spinal cord. At the C3 to C4 level, an epidural abscess was noted to be circumventing the spinal cord with an associated broad-based disc bulge resulting in complete effacement of the thecal sac and moderate compression of the underlying cord. At the C4 to C5 level, there was a right inferolateral prevertebral abscess extending through the right C4-C5 neural foramina, into the epidural fat, causing complete effacement of the ventral thecal sac and moderate compression of the cord. In addition, there was edema and enhancement of the C4-C5 and C5-C6 intervertebral disc spaces and the respective superior and inferior vertebral body endplates consistent with discitis and osteomyelitis (Figure 4).

**Figure 5:**
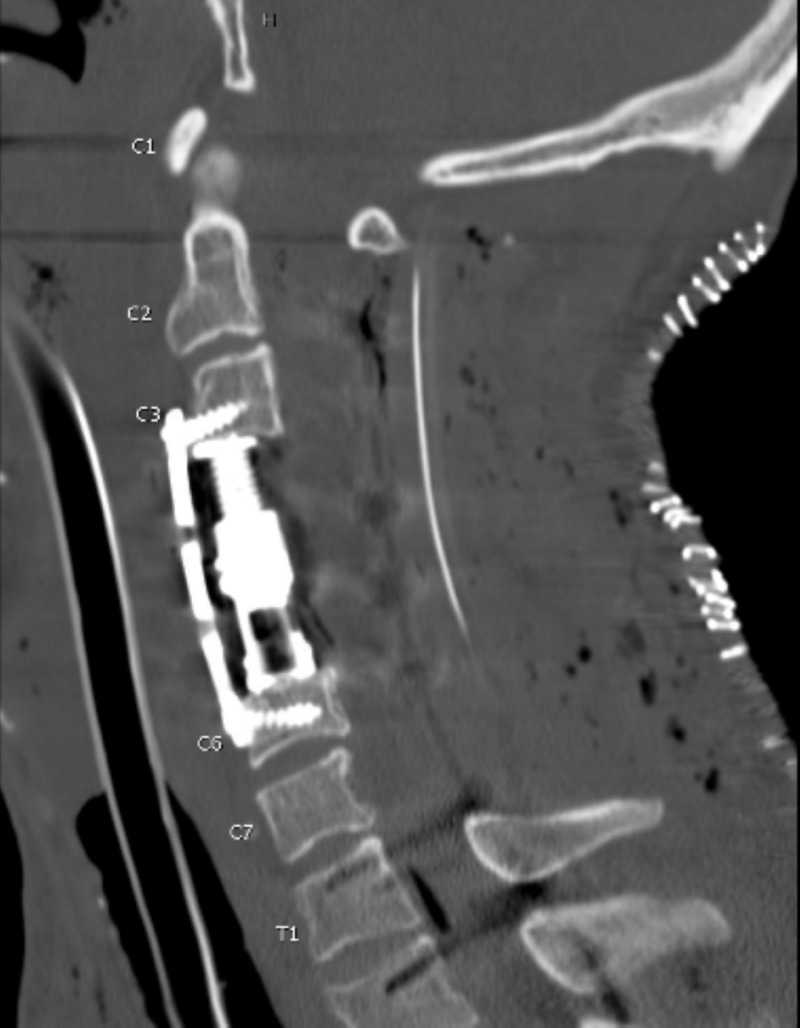
Post-operative sagittal CT non-contrast of the cervical spine Intubation tube is seen within the trachea. Postoperative changes including posterior drainage catheter and extensive subcutaneous emphysema are present. There is an anterior spinal fusion between C3 and C6 vertebrae with the corpectomy of C4-C5.

In the setting of significant neurological impairment in association with epidural abscesses extending from C1 to C5, systemic anticoagulation was discontinued and the patient was emergently taken to the operating room by neurosurgery for exploration and washout. Intraoperatively, an extensive purulent collection was noted within the anterior and posterior epidural space with a macerated dural defect and intradural spread. Due to an extensive spinal involvement, the patient ultimately underwent C4-C5 corpectomy, C2-T2 posterior decompression, and fusion along with evacuation of retropharyngeal and epidural abscess (Figure 5). The cerebrospinal fluid sample and multiple abscess fluid samples were sent for culture.

Blood cultures obtained at the time of arrival to the ED returned positive for S. intermedius growth. All intraoperative samples also grew S. intermedius. One intraoperative sample revealed polymicrobial growth that included S. intermedius, Streptococcus salivarius, Rothia mucilaginosa, and Rothia dentocariosa. Surgical pathology of the C4 and C5 vertebral tissue culture grew S. intermedius. The cerebrospinal fluid analysis showed pleocytosis, elevated protein, and decreased glucose but no organism growth. The infectious disease department was consulted at this time for antibiotic recommendations. Due to the growth of the same organism from blood and operative cultures originating from a deep neck space infection complicated by cervical epidural abscess and osteomyelitis in the setting of an IJV thrombosis, a diagnosis of LS secondary to S. intermedius was made. Culture sensitivity results eventually showed sensitivity to ceftriaxone and penicillin after which cefepime was converted to high dose intravenous ceftriaxone two grams every 12 hours with metronidazole 500 milligrams three times daily continued for anaerobic coverage. The decision to administer high dose ceftriaxone was made considering the possibility of meningeal infection. Repeat blood cultures drawn 48 hours later were negative. Of note, two-dimensional transthoracic echocardiography showed no evidence of cardiac valve vegetation.

**Figure 6:**
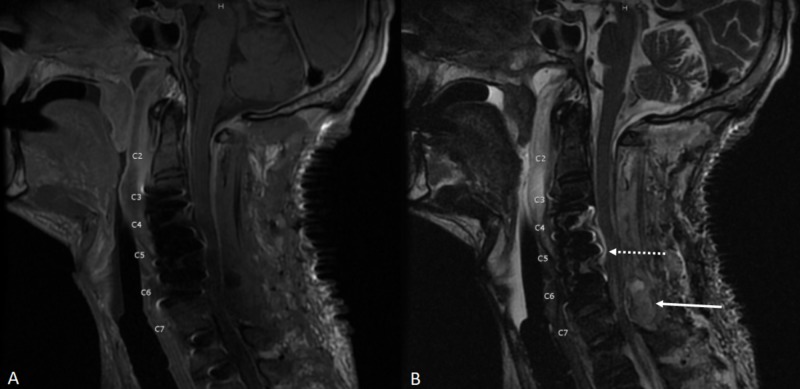
Sagittal MRI of the cervical spine (A) T1: Susceptibility artifact from the spinal fusion hardware limits evaluation and  (B) T2: Despite decompression, there is spinal canal stenosis with persistent mass effect and mild cord compression C4-5 (dashed arrow) and posterior cervical hematoma causing severe spinal canal stenosis and cord compression at C6-7 (solid arrow).

On postoperative day one, repeat MRI showed a persistent fluid collection with mass effect resulting in cord compression (Figure 6).

**Figure 7:**
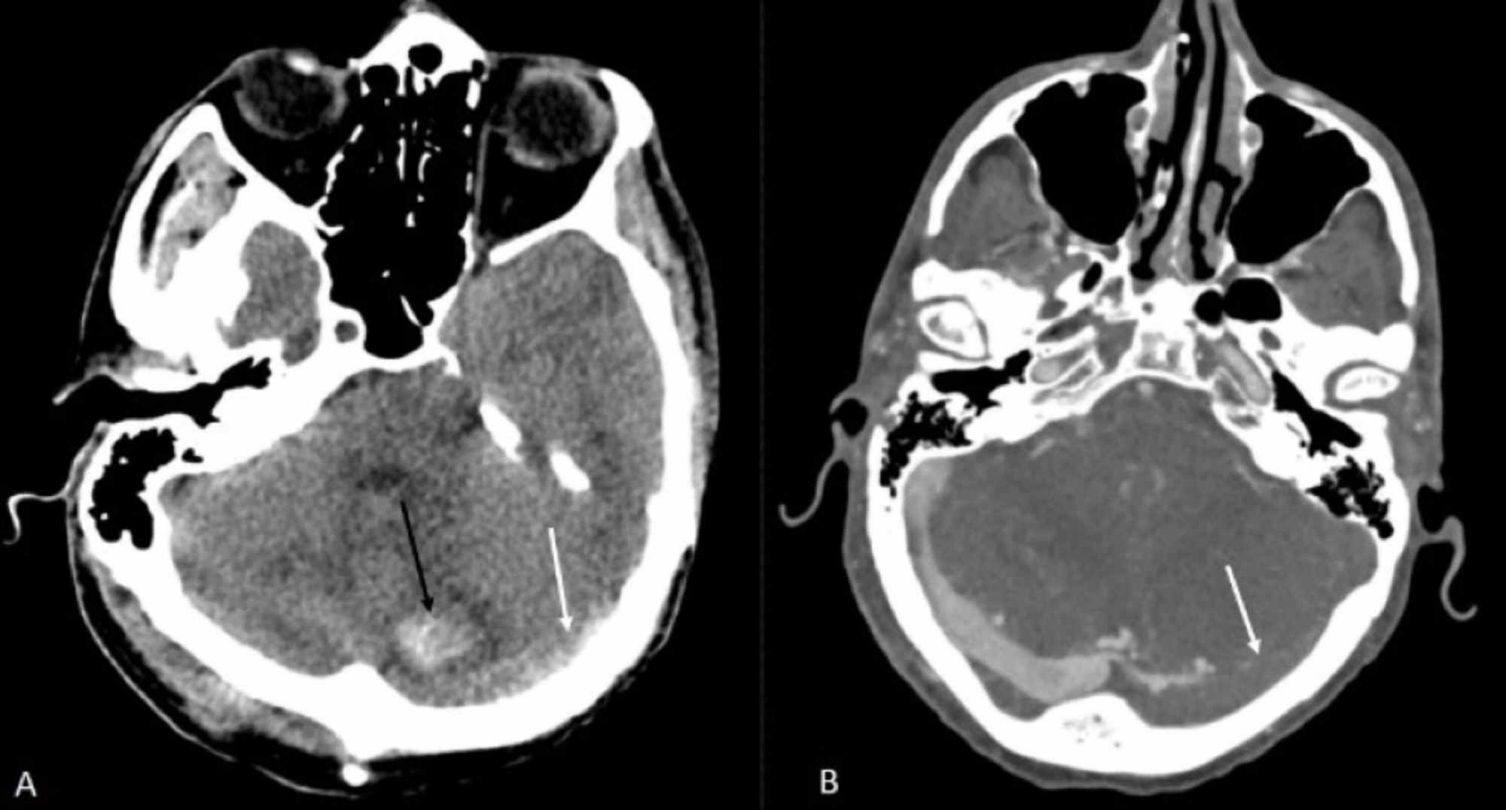
Axial non-contrast CT and CT venogram of head (A) Axial non-contrast CT head showing left cerebellar hemorrhage (black arrow) and a dense left sigmoid sinus consistent with sigmoid thrombosis (white arrow), (B) Axial CT venogram of the head showing non-opacification of the left sigmoid sinus (white arrow) and well opacified right sigmoid sinus.

The patient was emergently taken to the operating room for repeat exploration. During this exploration, a large posterior cervical epidural hematoma was discovered and evacuation of the epidural hematoma was performed. The following day, the patient developed worsening mental status and new-onset bilateral lower extremity weakness. CT venogram of the head performed at this time showed multiple sites of intracerebral hemorrhage as well as thrombus propagation from the left IJV and left sigmoid sinus into the left transverse sinus (Figure 7).

**Figure 8:**
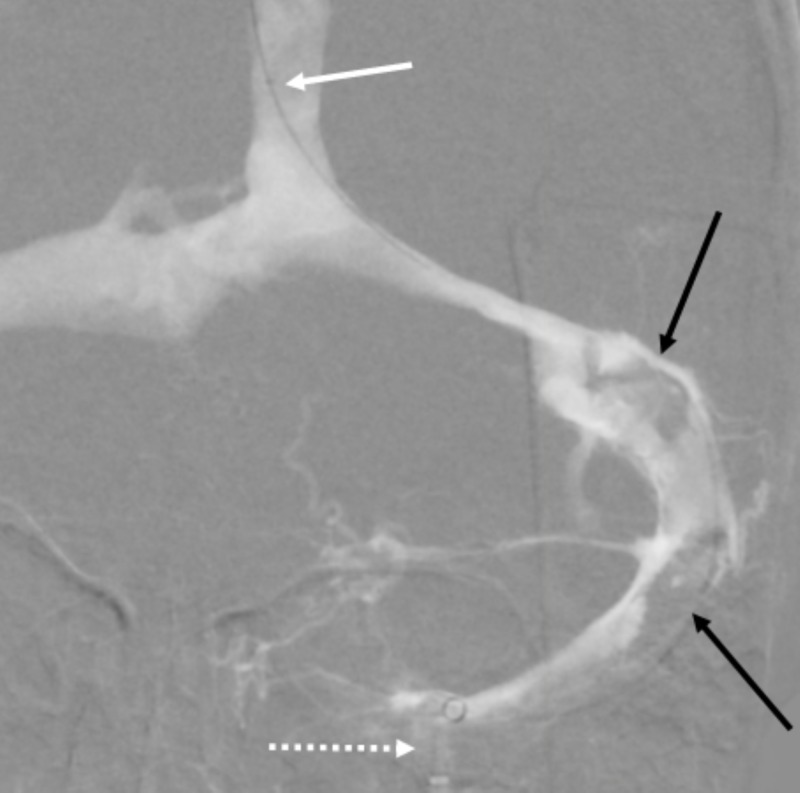
Coronal view of left jugular venogram status post mechanical thrombectomy Aspiration catheter in the left jugular bulb (dashed white arrow) demonstrating interval reperfusion of the left transverse, sigmoid and jugular systems with evidence of significant residual thrombus in the left sigmoid sinus (black arrows). Microcatheter placement in the left transverse sinus for possible intrasinus tPA administration (white arrow). tPA – tissue plasminogen activator

At this time systemic anticoagulation was contraindicated in the setting of recent surgery complicated by epidural hematoma. Due to progressive cerebral hemorrhage secondary to venous congestion, the patient underwent emergent diagnostic cerebral angiogram with left transverse sinus, sigmoid sinus, and IJV mechanical thrombectomy with microcatheter placement in the left transverse sinus for intrasinus administration of tissue plasminogen activator (tPA) (Figure 8).

Following this procedure, tPA was administered for 36 hours. In addition, the risks and benefits of systemic anticoagulation were weighed and the decision was made to initiate a low dose heparin drip. On day 3 post thrombectomy, the clinical course was complicated by a new dorsal epidural hematoma requiring emergent C2 to T2 wound re-exploration and evacuation of an epidural hematoma. Given the tenuous state of the patient's spinal cord, the heparin drip was discontinued.

**Figure 9:**
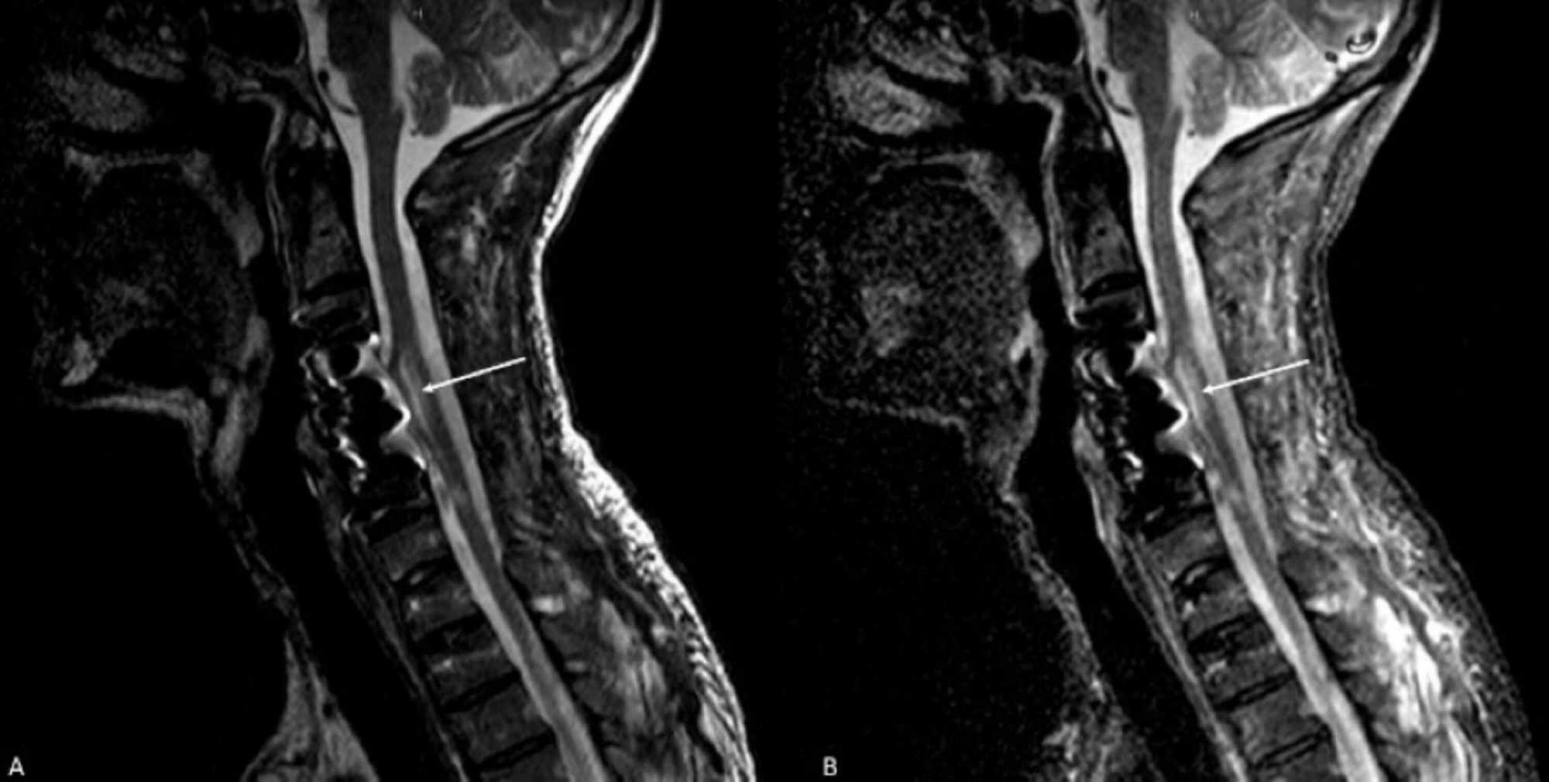
Sagittal MRI of the cervical spine Sagittal MRI of the cervical spine (A) T2 and (B) STIR. The spinal cord shows myelomalacia and cord atrophy at the C4-C7 (arrow). There is also deformity of the spinal cord along the ventral aspect, likely secondary to ventral adhesions of the cord with the metallic graft at the corpectomy site. STIR - short TI inversion recovery

The remainder of the hospital course was complicated by acute respiratory failure secondary to complete left lung collapse and extubation failure secondary to neuromuscular weakness requiring temporary percutaneous tracheostomy. However, the patient's neurological status improved thereafter and the fever and leukocytosis resolved. During the conclusion of a six-week course of intravenous ceftriaxone and metronidazole, the patient developed a skin rash consistent with drug eruption with biopsy showing evidence for leukocytoclastic vasculitis. The antibiotic course was converted to intravenous ertapenem for one week. MRI of the cervical spine following the full seven-week course of antibiotics showed spinal cord myelomalacia and cord atrophy from C4 to C7 without further evidence of fluid collection or cord compression (Figure 9).

At this time, the erythrocyte sedimentation rate (ESR) was noted to have decreased to 32 millimeters per hour, down from 41 millimeters per hour and the C-reactive protein (CRP) to 7.8 milligrams per liter, down from 208.9 milligrams per hour. Dental surgery was consulted during the hospital course who recommended multiple dental extractions in an outpatient setting. The patient was discharged to physical medicine and rehabilitation for further inpatient rehabilitation. At one year follow-up, the patient displayed a slow but persistent neurological recovery throughout an ongoing physical therapy regimen with mild residual weakness of his bilateral lower extremities, contractures of his right upper extremity, and chronic indwelling Foley catheterization. At this point, he has regained function to the extent that he is fairly independent with his activities of daily living.

## Discussion

S. intermedius is a rare causative organism for LS with only a few cases reported in the literature [8, 10-13]. Chemal et al. reported a case of a 21-year-old male patient with LS secondary to S. intermedius presenting with fever and lower chest pain related to multiple pulmonary abscesses subsequent to a recent pharyngeal infection [10]. Escalona et al. reported a case of a 36-year-old male with LS secondary to S. intermedius who presented with fever and extensive mandibular and floor of the mouth swelling due to an infected molar [11]. Gupta et al, reported a case of LS due to S. intermedius, likely secondary to gingival scraping, in which the presenting complaint was neck pain and persistent headaches. In this case, there was no fever and the patient had a benign oropharyngeal exam [8]. Cheta reported a 78-year-old male with LS due to S. intermedius who presented with eye and facial pain after a recent dental extraction. The hospital course was complicated by parapharyngeal and masticator abscesses [12]. Martel reported an 86-year-old male presenting with fever and left acute orbital syndrome due to pansinusitis and eventually septic thrombosis of the cavernous sinus that extended to the ipsilateral IJV and transverse sinus. Blood cultures, in this case, identified S. intermedius along with the Staphylococcus warneri species [13]. 

None of the cases of LS caused by S. intermedius described above were complicated by epidural abscesses although epidural abscesses have been reported secondary to other organisms. In 2006, Park et al. presented the first known case of LS with an epidural abscess in a 43-year-old woman who presented with acute confusion and bilateral lower extremity weakness, preceded by a pharyngeal infection. In this case, F. necrophorum was found in the enhanced cultures using the polymerase chain reaction [14]. Eight cases of LS with epidural abscesses have been reported since that time. F. necrophorum was identified as the etiological agent in six out of those eight cases [7]. In 2018, Shimamoto et al. described a case of LS in a 55-year-old woman with an epidural abscess and retropharyngeal abscess due to Staphylococcus aureus [15]. Sabaka et al. in 2019 described the case of a 19-year-old male with LS secondary to Klebsiella pneumoniae, complicated by an epidural abscess [7]. 

Most reported cases of LS occur during the second decade of life (51%), followed by the third decade (20%), and then by the first decade. Sore throat is the most common presenting symptom followed by symptoms of IJV thrombophlebitis such as neck pain and neck mass, followed by symptoms of complications and metastatic infections. Fever is the most common physical finding reported, followed by pharyngitis or peritonsillar abscess and neck mass, then followed by cervical lymphadenopathy [2, 4]. Central nervous system complications are rare but include the retrograde extension of thrombus into the cavernous sinuses, meningitis, and cerebral abscesses. Epidural and subdural involvement is rarely reported as described above [16]. The initial symptoms of fever, headache, neck pain and difficulty in swallowing in our patient were likely related to the presence of retropharyngeal and neck abscesses, and IJV thrombosis. Although the presenting neurological symptoms in our patient were mild, his neurological status rapidly deteriorated over the first two days of admission, from mild right upper extremity weakness and acute urinary retention to overt changes in the sensorium and bilateral lower extremity weakness. This could be explained by progressive cerebral venous infarction due to the propagation of the left IJV thrombosis into the ipsilateral sigmoid sinus and left transverse sinuses which resulted in multiple sites of intracerebral hemorrhages.

A diagnosis of LS can be considered based on a history of a recent pharyngeal infection, clinical or radiographic evidence of thrombophlebitis of the internal jugular vein (IJV), and isolation of an anaerobic pathogen from blood culture [8]. In regards to imaging studies, a CT scan of the head and neck with intravenous contrast is considered diagnostically superior to a neck ultrasound as it is better in locating the anatomical extent of thrombosis. MRI is an excellent method for visualizing all anatomic structures as well as the extent of thrombosis and/or septic embolization. In our patient, it was likely that in the setting of the poor dentition and severe tooth decay, an episode of seizure resulted in translocation of bacteria into the retropharyngeal space which locally invaded the parapharyngeal space causing septic thrombophlebitis of the IJV and subsequent S. intermedius bacteremia. In the modern era, prolonged antibiotic treatment and surgical exploration and debridement, if necessary, are considered as the mainstay of treatment for LS [4]. Surgical treatment of LS may involve drainage of abscesses of the neck, lungs, liver, and epidural space. The peritonsillar and lateral pharyngeal spaces are the most common locations in the head and neck requiring abscess drainage [2]. Our patient underwent emergent surgical debridement due to the extent of neurological symptomatology in the presence of spinal cord compression and epidural abscesses. The suggested empirical antibiotics for treatment of LS include clindamycin, carbapenems, beta-lactam/beta-lactamase inhibitors, or a combination of penicillin plus metronidazole, tailored to cultures and sensitivity. Some authors recommend against the use of monotherapy with metronidazole due to the frequent occurrence of a mixed infection with other oral flora. The duration of antibiotic therapy should be from two to six weeks [2, 4, 6]. In our patient, although S. intermedius was the predominant pathogen, one intraoperative culture grew Rothia species and Streptococcus salivarius in addition to S. intermedius, further corroborating an odontogenic source. Because of this, anaerobic coverage with metronidazole was included along with an increased dose of intravenous ceftriaxone in the setting of intradural involvement and the possibility of meningitis. During the sixth week of treatment, the antibiotic regimen required conversion to intravenous ertapenem following drug eruption.

Anticoagulation remains a controversial issue in patients with LS and no controlled studies exist. Case series have reported that between 21% and 30% of patients are treated with anticoagulation. Some have advocated for the use of anticoagulants in all cases of LS while others have recommended anticoagulation only in circumstances where IJV thrombosis extends into the cerebral sinuses and symptoms fail to improve with antibiotics alone [2, 4, 6, 17]. Obviously, anticoagulation is associated with an increased risk of bleeding and hematoma expansion. In our patient, a low dose anticoagulation regimen was associated with the formation of a large epidural hematoma which required reoperation twice for evacuation and which precluded further administration of anticoagulation. In the pre-antibiotic era, intravenous vein ligation and excision were frequently performed to prevent septic embolization, but this is rarely performed in this modern era [2, 4, 6]. In our patient, endovascular management via left transverse sinus, sigmoid sinus, and IJV mechanical thrombectomy with intrasinus administration of thrombolytic agent was performed in the face of a major contraindication to anticoagulation and was carried out successfully.

## Conclusions

In conclusion, we report a case of LS caused by S. intermedius associated with the rare complication of cervical epidural abscess. LS has been regarded as a forgotten condition in the post-antibiotic era, but seems at this point to be a re-emerging condition. Additionally, the case described above highlights the well-known virulence of S. intermedius for abscess formation. LS continues to be a potentially life-threatening syndrome associated with grave complications but aggressive measures including intravenous antibiotics and surgical intervention can lead to a favorable outcome.
